# Nonlinear metamaterials for holography

**DOI:** 10.1038/ncomms12533

**Published:** 2016-08-22

**Authors:** Euclides Almeida, Ora Bitton, Yehiam Prior

**Affiliations:** 1 Department of Chemical Physics, Weizmann Institute of Science, Rehovot 76100, Israel; 2 Department of Chemical Research Support, Weizmann Institute of Science, Rehovot 76100, Israel

## Abstract

A hologram is an optical element storing phase and possibly amplitude information enabling the reconstruction of a three-dimensional image of an object by illumination and scattering of a coherent beam of light, and the image is generated at the same wavelength as the input laser beam. In recent years, it was shown that information can be stored in nanometric antennas giving rise to ultrathin components. Here we demonstrate nonlinear multilayer metamaterial holograms. A background free image is formed at a new frequency—the third harmonic of the illuminating beam. Using e-beam lithography of multilayer plasmonic nanoantennas, we fabricate polarization-sensitive nonlinear elements such as blazed gratings, lenses and other computer-generated holograms. These holograms are analysed and prospects for future device applications are discussed.

## Abstract (web summary)

Phased metasurfaces have been extended to the nonlinear regime, enabling coherent generation, beam steering and lensing of light beams in one thin element. Here, Almeida *et al*. demonstrate a nonlinear multilayer metamaterial hologram generating images at the third harmonic frequency of the illuminating beam.

## Graphical abstract

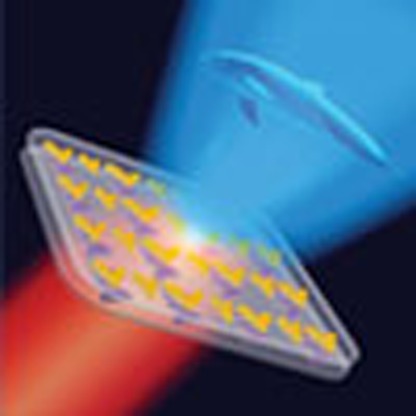

Holography was invented many decades ago^1^,^2^ and was rapidly recognized as an effective means for storing and reconstructing images. By interference of a beam coming from the object and a reference coherent (laser) beam, phase and possibly also amplitude information has been stored in some media, typically a film that responds to the intensity of the light falling on it. The holographic method was developed to correct spherical aberration of electron lenses, but following the invention of the laser, optical holography^2^,^3^ overshadowed the field of holography, eventually leading to applications such as volumetric data storage^4^, optical tweezers^5^ and three-dimensional (3D) displays^6^. Other kinds of waves were also used in non-optical holography, such as electron gas quantum holography^7^ and plasmon holography^8^.

In recent years, new storage elements have been proposed^9^,^10^,^11^ using optical metamaterials, which consist of (metallic) nanoantennas that typically encode phase information for reconstructing images (phase holograms). Metamaterials are periodically nanostructured artificial materials, which can manipulate light–matter interactions on spatial dimensions smaller than or comparable to the wavelength of light^12^. The optical response of these metamaterials can be engineered to exhibit unusual optical phenomena such as negative refraction^13^,^14^ or optical cloaking^15^, and can lead to novel applications. Metasurfaces are ultrathin, quasi-two-dimensional metamaterials made of metallic or dielectric nanostructures, which can locally control the phase, amplitude or the polarization state of light waves propagating through or reflected from them^16^,^17^,^18^,^19^,^20^,^21^. Such control is the working principle behind their application as holographic metasurfaces^22^, and 3D metamaterials^23^,^24^ were also used^9^. So far, the vast majority of metasurfaces, with a few exceptions^25^,^26^,^27^, have been designed to operate in the linear regime. Therefore, while they shape the wavefront of light, they cannot alter its frequency. More recently, the concept of phased metasurfaces was extended to the nonlinear regime^28^,^29^,^30^,^31^, enabling both coherent generation and manipulation such as beam steering and lensing of light beams. Nonlinear phase control has been demonstrated for second harmonic generation in arrays of metallic split-ring resonators^28^, third harmonic generation (THG) in cross-shaped metallic nanoparticles for circularly polarized light^29^ and four-wave mixing in metallic thin films^32^.

Plasmonic metasurfaces^9^,^10^,^11^ have been used for computer-generated holograms^33^,^34^, which were further used for various applications^35^, where a target image is digitally processed and the phase pattern of the hologram is calculated using numerical methods for light propagation/diffraction. The image is reconstructed by a reading laser beam that illuminates the storage medium. In standard computer-generated holography, the image is formed at the same wavelength of the reading laser beam.

Here we build on the concept of nonlinear phase control in plasmonic metasurfaces, and demonstrate THG holograms where the image is formed at a wavelength different from the reading beam, as illustrated in Fig. 1. We demonstrate computer-generated holograms stored in multilayered plasmonic metasurfaces and exhibit polarization as an additional control parameter. The high-density storage enabled by sub-micron nanoantennas, especially in multilayered structures, may lead to holograms with very high resolution.

## Results

### Antenna design

**Figure 1:**
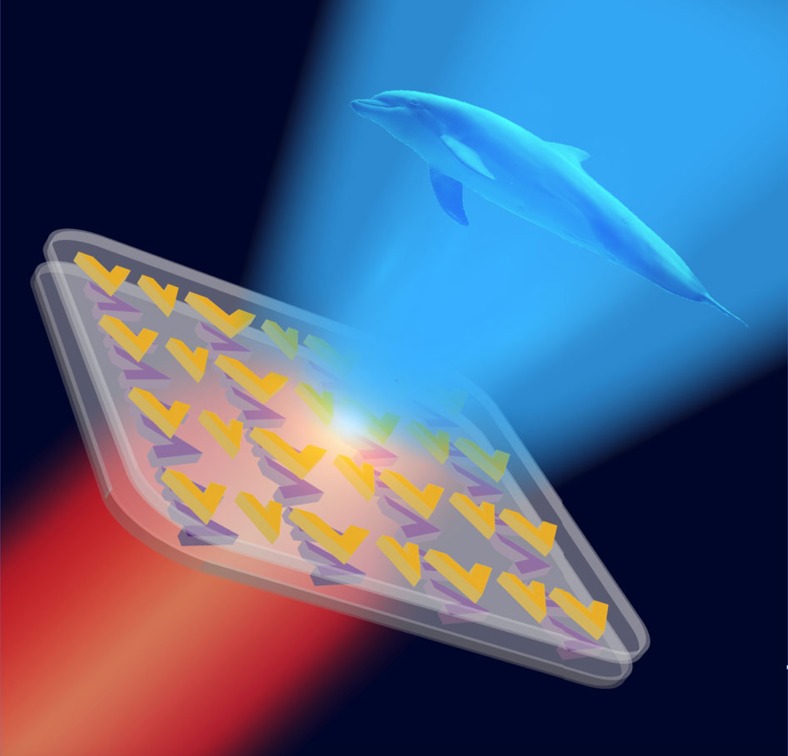
Artist's concept of a multilayer nonlinear metamaterial hologram. When the hologram is illuminated by an infrared laser, it generates a holographic image of an object at the third harmonic frequency in the blue.

**Figure 2:**
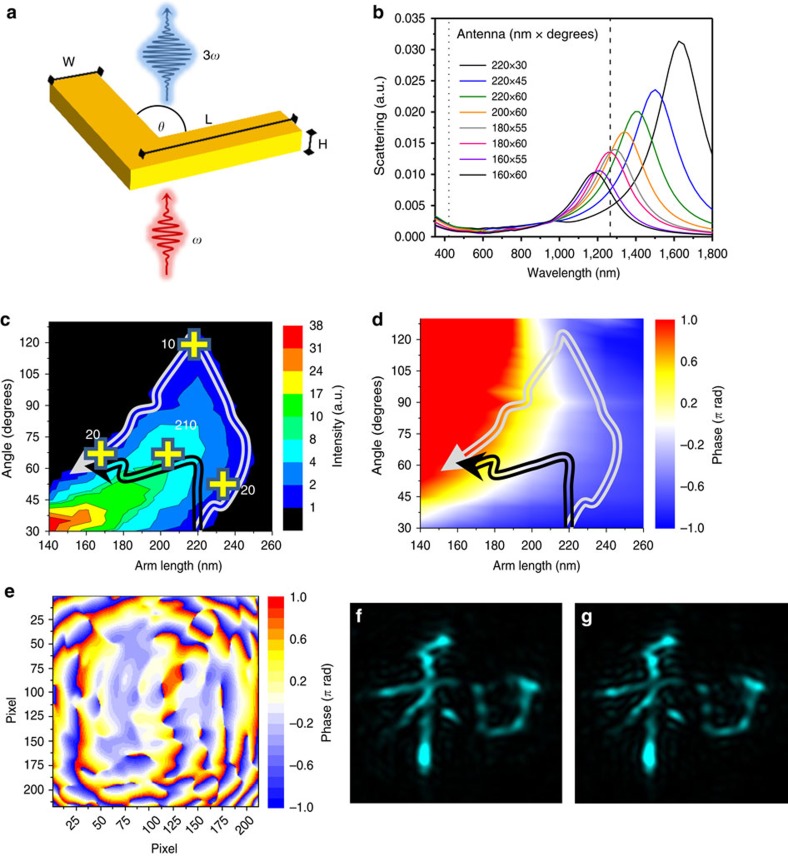
Design of phase-controlled nonlinear nanoantennas. (**a**) Dimensions of the V-shaped gold antennas for local control of the phase of the nonlinearity. The free parameters are the arm length *L* and the angle *θ* between the arms. *H*=30 nm and *W*=40 nm are constant for all antennas. (**b**) Representative linear scattering spectra of some of the antennas (*L* × *θ*), the input light was polarized along the bisecting axis of the V-shaped antennas. The dashed and dotted lines indicate the free-space wavelengths of the fundamental (*λ*_F_=1,266 nm) and third harmonic (*λ*_F_=422 nm) beams respectively. Intensity (**c**) and phase (**d**) map of the third harmonic signal with two possible paths: the white path with minimal amplitude variations and the black path, with larger amplitude variations but with much larger extinction for the ‘wrong' polarization (see Supplementary Note 2). The (+) crosses indicate the extinction ratio of third harmonic signal for two orthogonal polarization modes. (**e**) Depicts the calculated phase distribution on a sample hologram (see below) and the corresponding numerical reconstruction for antennas chosen along the black path (**f**) for a distance of 750 μm. (**g**) Shows the same hologram but calculated for a constant amplitude with hardly any differences observable.

To generate holograms at the new frequency, phase information of an object is encoded in a set of computer-generated nanoantennas in the multilayer metamaterial. After illumination with a suitably polarized infrared laser, the image of the object is reconstructed at a frequency that is the third harmonic of the incoming beam. The general configuration is visualized in Fig. 1. Metallic nanostructures are attractive candidates for efficient harmonic generation due to the high nonlinearities of metals^36^, and the near-field enhancement at plasmonic resonances, which can boost frequency conversion by orders of magnitude^28^. As our basic nanostructured element, we chose linearly polarizable V-shaped gold antennas as depicted in Fig. 2a. An alternative choice could have been metallic rods of varying aspect ratio and orientation angle. Several factors were evaluated: the spectral response of the individual antennas, the polarization response (that is, the extinction ratio of the ‘wrong' input polarization) and the strength of the nonlinear THG response. Since our goal was to fabricate several non-interacting layers, the V-shaped antennas offer superior performance in that the spectral response of the ‘wrong' polarization is spectrally removed from both the fundamental and the THG frequencies, and the extinction of the 'wrong' polarization is very high. This point is elaborated on in Supplementary Figs 1 and 2 and discussed in Supplementary Note 1.

For symmetric V-shaped antennas, the two parameters that we change are the length of the arms and the angle between them. As these parameters are changed, the plasmonic resonances are tuned across the near-infrared spectrum. Near resonance, the electronic cloud of the nanoantennas is driven by a phase-shifted effective field 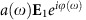, where **E**_1_ is the incoming light field, *a*(*ω*) is the near-field enhancement and *ϕ*(*ω*) is the shape-dependent phase-shift imposed on the incoming beam. Approximating the nonlinear nanoantennas as point dipoles of effective third-order nonlinear susceptibility 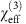, the third-order polarization (oscillating at 3*ω*) induced in the dipole is given by


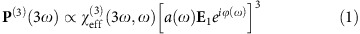


Therefore, if *ϕ*(*ω*) is the phase-shift of the fundamental beam, the phase shift to the third harmonic field **E**_3_ will be 3*ϕ*(*ω*) where an additional relative phase shift may be added^31^ depending on the resonant nature of 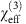. Figure 2 provides the information about the antennas and their phase control. Figure 2a depicts the antenna and the dimensions of the relevant parameters (arm length and angle); Fig. 2b depicts finite differences time domain (FDTD) calculations of the linear plasmonic resonance transmission intensity for various combinations of arm lengths and angles. It is clearly seen that the resonance peak moves to longer wavelengths for longer arm length and smaller angles.

To generate our phase holograms, which follow a working principle similar to the earliest kinoform-type holograms^37^, we need phase information for each element at the THG wavelength. The maps of amplitude and phase of the THG generated at *3ω*=422 nm , calculated by nonlinear FDTD, are presented in Fig. 2c,d. We used a realistic range of parameter that is enough to generate a 2π relative phase shift when both the fundamental and the third harmonic beams are of the same polarization. A path along which to design the phase elements is a guide for the choice of parameters. Two such paths are depicted in Fig. 2c,d. The white path provides small variation of the intensity, but at the same time, low extinction for the perpendicular polarization, and low field enhancement of THG. The black path, on the other hand, provides the same 2*π* phase range, much higher field enhancement and excellent extinction ratio for the ‘wrong' polarization. Moreover, the white path involves elements with opening angles as large as 120°, which necessitates larger separation distance between the individual elements, leading to lower density of elements, namely reduced efficiency. The price for using the black path is a somewhat larger intensity variation, but as we show, this variation does not affect the fidelity of the holograms—they are as good as ‘pure' phase holograms, but with the higher enhancement for the THG response. The selection of the optimal path and the effects of the intensity variations are discussed further in Supplementary Figs 2–5 and Supplementary Note 2.

To test the concepts introduced above, we started with a very basic setup of a two-layer polarization sensitive nonlinear blazed grating, where each layer was designed to diffract a different polarization. Since these V-shaped nanoantennas are polarization specific and do not show any plasmonic resonances at the third harmonic frequency, the phase is not changing on propagation through the sample, thus enabling us to introduce multilayer structured phase holograms. The details are given in Supplementary Fig. 6 and in Supplementary Note 3.

### Sample fabrication

The samples are prepared by multilayer e-beam lithography on a borosilicate glass substrate. A 180 nm thick silica layer is deposited by plasma-enhanced chemical vapour deposition (PECVD) on the silica substrate. The desired design of 30 nm thick antennas is patterned into the silica layer by e-beam lithography and dry etching. The process is then repeated for additional layers where the deposited silica layer serves as a dielectric spacer between two adjacent active nanoantenna layers. The subsequent layers are stamped with high spatial accuracy (of order 10–20 nm). While this accuracy is not necessary for the present work, it will be important and even critical for future implementation of the technique currently under investigation. Details are given in the ‘Methods' section and in Supplementary Methods.

### Computer-generated metamaterial holograms

Several different types of metamaterial holograms were fabricated; all of them nonlinear where the wavelength of the illuminating input beam is 1,266 nm and the images are formed at the third harmonic at 422 nm. Different polarizations were utilized for different images, and each image was encoded into a single layer in a multilayer composite metamaterial hologram. In some cases (not shown here), more than one polarization coded image was stored in a layer. Some of the holograms were designed such that the each image was formed at a different distance, thus laying the foundation to 3D capabilities.

**Figure 3:**
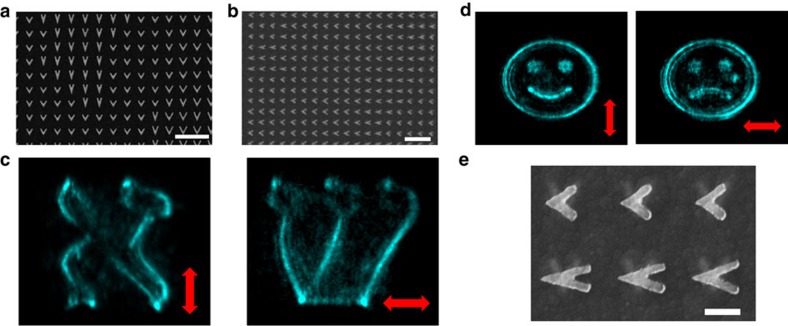
Nonlinear metamaterial holograms. Scanning electron microscopy (SEM) images of the first (**a**) and the second layer (**b**) of a 3D nonlinear hologram, which projects holographic images of the Hebrew letters Alef and Shin (**c**) for vertically or horizontally polarized input beams, respectively. (**d**) Nonlinear holograms of smiley and sad faces for vertically or polarized fundamental beams respectively. (**e**) SEM image of two typical overlaid layers of nanoantennas. Scale bars, 1 μm (**a**,**b**), 200 nm (**e**).

Figure 3 depicts two different double layer structures with different holograms embedded in each layer for each structure. For each layer within the multilayer metamaterial, a separate phase hologram was computer generated to yield the desired far-field image for the proper polarization. For one structure, the Hebrew letters Aleph and Shin are generated for vertical and horizontal polarizations respectively and for the second, images of happy and sad smiley faces are generated. Note that each image is recreated only by the properly polarized input beam, and emanates from a single layer of nanoantennas within the metamaterial. Figure 3e depicts an enlarged section of the multilayer structure, demonstrating the stamping accuracy of the different layers.

These nonlinear phase holograms work in transmission and at the THG frequency there is no background noise as is the case for linear transmission holograms where the image is at the same frequency as the illuminating beam.

**Figure 4:**
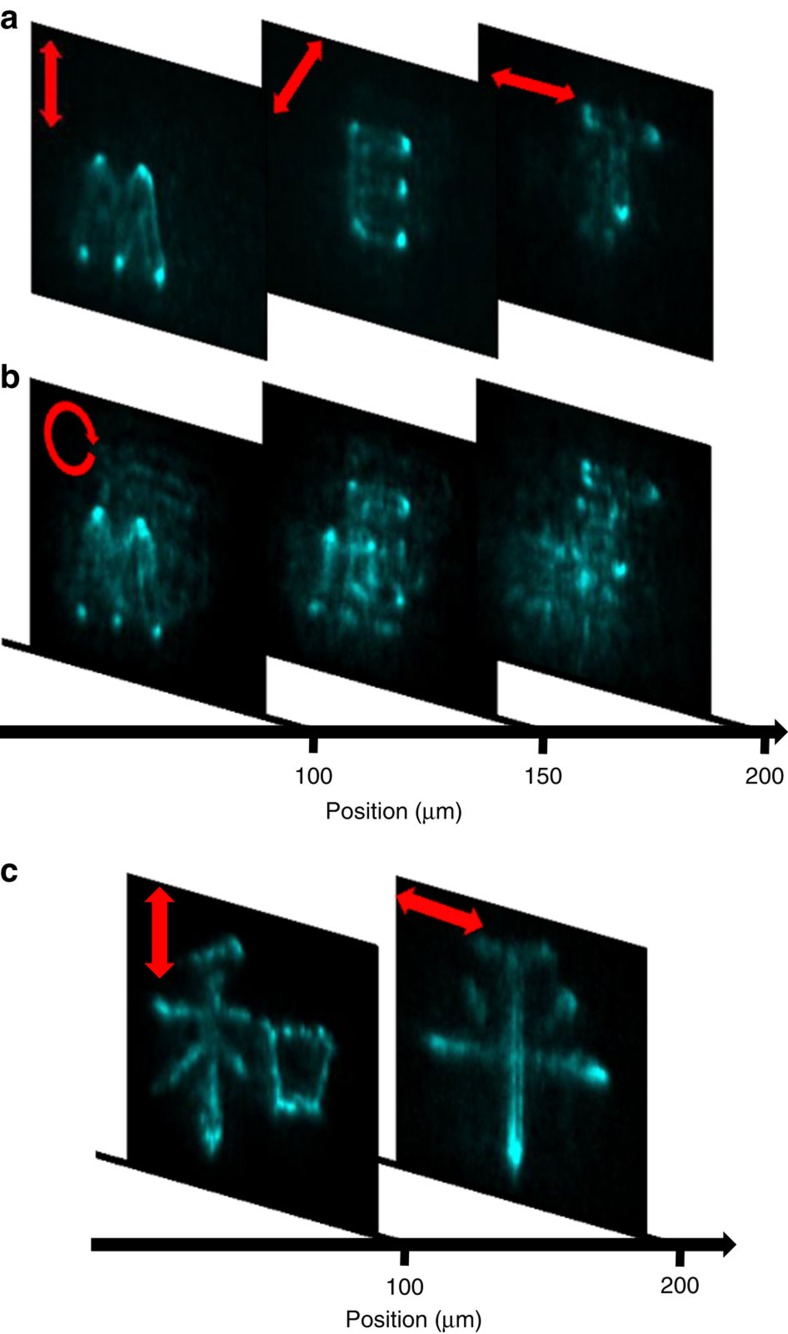
Nonlinear holograms displaying three-dimensional features. (**a**) Nonlinear (NL) images of a three-layer hologram displaying the word MET at different planes for light polarized at 0, 45 and 90 degrees. (**b**) NL images for input circularly polarized light. In a given plane, all the letters are visible but two of them appear unfocused. (**c**) Chinese characters for ‘Peace and harmony'.

The next two examples of polarization-dependent holograms depict images formed at different focal distances, three such distances in one case, and two in another. We computer generated a three-letter hologram MET (short for METamaterial), where each of the three letters was designed to be generated at different focal distance and for a different polarization (0°, 45° and 90°, respectively). The result is depicted in Fig. 4. To further illustrate the sensitivity to the correct polarization, images generated with the ‘wrong' polarization at the correct focal distance are also shown.

The last in this series of demonstrated metamaterial holograms are the Chinese characters for ‘Peace and Harmony' (Fig. 4c), where greater detail and better accuracy are required. The images are recreated by the proper polarizations at different focal distances.

In Supplementary Fig. 7, we demonstrate how different polarizations give rise to three different but related images; and in Supplementary Movie 1, the continuous scanning of the input polarization creates a dynamic hologram that gives the impression of moving wings of a fan.

**Figure 5:**
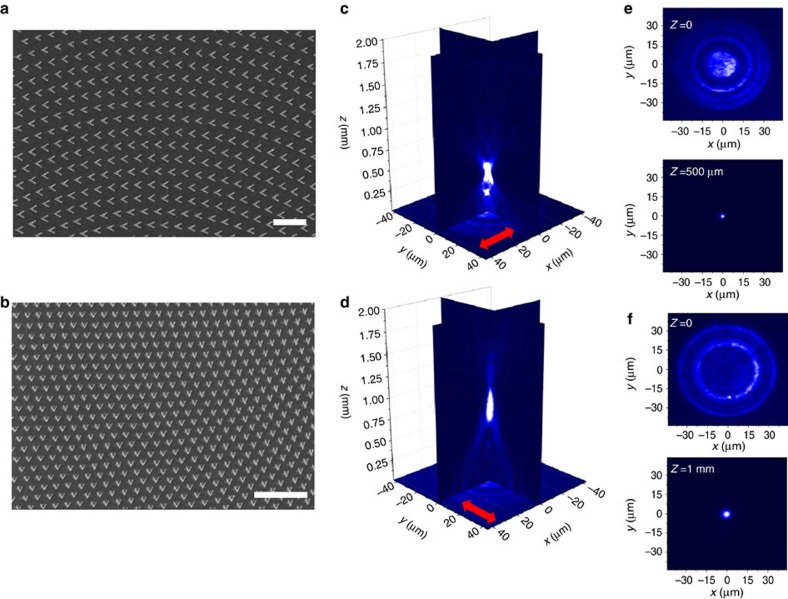
A polarization-controlled nonlinear lens. Scanning electron microscopy (SEM) images of the first (**a**) and the second (**b**) layers of a nonlinear lens made by a 3D nonlinear metamaterial. The scale bars are 1 and 2 μm in **a** and **b**, respectively. Microscopic images of the third harmonic signal away for the surface of the metamaterial device for a horizontally (**c**) or vertically (**d**) polarized fundamental beam. The first active layer (**a**) focuses the TH radiation around the focal point *z*=500 μm (**c**), while the second layer focus the light at *z=*1 mm (**d**). Microscopic images at *z*=0 and *z*=500 μm for horizontal polarization (**e**) and *z*=0 and at *z=*1 mm for vertical polarization (**f**). The Gaussian RMS widths at *z*=500 μm and *z=*1 mm are *σ*=1.2 and 2.2 μm, respectively, close to the theoretical diffraction limited values *σ*=1.1 and 2.2 μm.

The direct implementation of the ability to steer the beam in a polarization-dependent manner is manifested in the design of nonlinear metalenses. A nonlinear Fresnel-like multilayer metalens focuses the TH radiation into two different focal points depending on the input polarization of the fundamental beam (Fig. 5). The phase profile necessary to obtain light focusing is given by 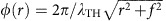 here λ_TH_ is the free space wavelength of the third harmonic beam and *f* is the focal distance. The metamaterial focuses the third harmonic at *f*=1 mm for vertical polarization and at *f*=500 μm for horizontal polarization into nearly diffraction-limited spots. The lenses are intrinsically nonlinear and work only for the signal that is coherently generated *in situ*. An incoming beam of the frequency identical to that of the THG beam will not be focused, nor is the incoming fundamental beam focused by this unique lens. The actual beam propagation through the focal regions of such metalenses with focal distances of 1 and 0.5 mm are shown in Supplementary Movies 2 and 3.

## Discussion

The ability to design and fabricate nonlinear metamaterials with local control of the nonlinear phase in all spatial dimensions enable the realization of efficient functional nonlinear devices with additional degrees of freedom and may have applications in volumetric data storage (write only). In this work, we demonstrated unique polarization multiplexed holograms that are generated at a new wavelength (the THG) and therefore background free. In spite of some amplitude variations, these are phase holograms, and it is clearly shown that the amplitude changes are not harmful to the performance of the holograms. Naturally, with this method, information may also be embedded in the amplitude of each nanometric element. While in principle flat metasurfaces can also provide polarization sensitivity, in our case, the volumetric, multilayer metamaterials enable higher density, which is crucial for the realization of efficient nonlinear devices. Moreover, since the individual element can be much smaller than the optical wavelength, the maximum data density can be higher than with standard films where the resolution is limited by optical diffraction.

The holographic method demonstrated in this work differs from the conventional optical holograms in the sense that in the present case the image is formed at a different frequency. This enables the realization of transmission holograms with low background. By changing the input polarization, different images are revealed, either at the same plane or at different planes, thus enabling the formation of dynamic 3D images, which are ‘moving' in response to changing polarization.

In the construction of the multilayer metamaterials, we were able to achieve high overlay accuracy of 10–15 nm. This accuracy is not really necessary for most of the holograms demonstrated in this work with the exception of the lenses, where some overlay accuracy, albeit lower, is needed. However, in anticipation of future work where true 3D nonlinearities, involving elements from different layers, are being designed, we have elaborated on our fabrication methodology and included evidence for this potentially very useful technological capability.

The efficiency of harmonic generation by metal-based metamaterials is lowered by metallic losses. Furthermore, higher input intensities may not be used due to the low optical damage threshold. In our optical elements, the conversion efficiency is of the order of 10^−8^, which surpasses the theoretical efficiency of a BBO crystal of equivalent thickness^32^. Further improvement can be attained at low input power by using hybrid metal–semiconductor structures, which is based on enhanced nonlinearities of intersubband transitions of semiconductor heterostructures^38^. However, high efficiencies are yet to be demonstrated in the visible spectral range. For higher power applications, an alternative approach is to use low loss semiconducting or dielectric CMOS-compatible materials such as silicon^39^, which has nonlinear refractive index *n*_2_=2 × 10^−14^ cm^2^ W^−1^ or silicon nitride^40^, which is transparent in the visible and is particularly attractive for three-dimensional metamaterials. The extension of the methods used in the present work to non-metallic structures is underway, based on the nonlinear numerical calculation of Mie resonances in dielectrics.

In summary, we demonstrate a method of volumetric storage of optical information using nonlinear 3D metamaterial devices. By encoding the nonlinear phase in the nanometric antennas, we build computer-generated polarization-sensitive nonlinear holograms as well as optical elements, such as lenses and blazed gratings. In this work, we converted an infrared incoming beam to a visible-range image, but the concept remains the same for other spectral ranges as well. These high-density nonlinear holographic 3D metamaterials may find applications as data storage devices and integrated nonlinear photonics.

## Methods

### Numerical simulations

The linear and nonlinear optical responses were calculated by the FDTD method using the commercial software Lumerical FDTD Solutions^41^. In all the simulations, the dimensions of the mesh around the nanoantennas were set to *dx*=*dy*=*dz*=5 nm, and perfectly matched layers were added in all dimensions to avoid reflections. The optical constants of Au and Cr were extracted from the CRC tables, while the optical constants of SiO_2_ were taken from Palik^42^. The linear scattering of the nanoantennas was calculated using a total-field/scattered field source. The third-order nonlinear susceptibility of gold was set to χ^(3)^=10^−19^ m^2^ V^−2^. The nonlinear light source was a linearly polarized transform-limited 60 fs long pulse centred at 1,266 nm with amplitude 5 × 10^8^ V m^−1^. The complex electric field of the third harmonic signal emitted in the forward direction is recorded on a *y*-normal plane, from which we extract the relative phase of the third harmonic signal. The amplitude of the THG is calculated by integrating the *z* component of Poynting vector on a power detector placed in the far field.

### Sample fabrication

The multilayer samples were fabricated using e-beam lithography. A 150 μm thick microscope cover slip was used as the substrate. To avoid electron charging on the substrate, a 3 nm thick chromium layer was initially deposited on the substrate by e-beam evaporation. A 180 nm silica layer was deposited by PECVD on top of the Cr film. The patterns were exposed by e-beam lithography (RAITH e_Line Plus) on a 125 nm thick resist (950 k PMMA A2). We used accelerating voltage of 30 kV and a beam current of 30 pA. Alignment marks were exposed at every e-beam exposure step and these were used for aligning the pattern of the next (top) layer. By using alignment marks, we achieved overlay accuracy between each two adjacent layers as high as 10 nm. After the sample was developed in MIBK:IPA (1:3), the silica layer was etched by inductively coupled plasma (SPTS). We used CF_4_ gas, 50 s.c.c.m. flow, 400 W inductively coupled plasma power and 50 W platen power. Etch time of 20 s results in ∼30 nm etch depth in the SiO_2_ layer. A 2 nm thick chromium adhesion layer and a 30 nm thick gold layer were then e-beam evaporated on the etched patterns. The resist is then lifted off in acetone leaving metallic patterns stuck in the SiO_2_ layer. A 180 nm thick silica layer is deposited again on the metallic pattern by PECVD. The whole fabrication process is repeated for the additional layers. The total lateral size of the structured sample is 80 μm and to minimize near field coupling, the centre-to-centre distance between the nanoantennas was maintained to be 410 nm. Further information is provided in Supplementary Methods.

### Nonlinear measurements

An optical parametric amplifier pumped by an amplified Ti:Sapphire laser was used as the light source. The optical parametric amplifier delivered 60 fs long pulses, centred at 1,266 nm and with repetition rate of 1 kHz. An achromatic half-waveplate (Casix, WPZ1315) was used to rotate the polarization of the beam. The fundamental beam was weakly focused on the sample by a 1 m lens to a waist of ∼100 μm. The average power was set to 380 μW. The TH signal was collected by an objective lens of numerical aperture 0.44 and filtered by bandpass and shortpass filters (Semrock, brightline 405/150, Semrock EdgeBasic SWP785, Thorlabs FESH0750). The images of the holograms and the lenses were projected onto an EMCCD camera (Andor, iXon^EM^+ 885) using another lens of focal distance 200 mm. A flip mirror was used to switch the signal between the EMCCD and a spectrometer (Jobin-Yvon, Triax 180 coupled to a CCD, Symphony, Jobin Yvon) for spectral analysis. The k-space projection of the nonlinear blazed grating was done by imaging the back focal plane of the objective onto the CCD using a 50 mm lens.

### Hologram design

The computer-generated phase holograms were designed using the point source method. In this method, each pixel 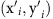 in the two-dimensional ‘object' is a point source of spherical light waves and the phase element at the hologram is calculated as the linear superposition of the electric fields emitted by all the point sources of the image. The holographic image is projected on the CCD camera and digitally inverted to obtain the image as seen by an observer looking from the sample to the image.

### Data availability

All relevant data are provided in the Supplementary Material and available with the authors.

## Additional information

**How to cite this article:** Almeida, E. *et al*. Nonlinear metamaterials for holography. *Nat. Commun.* 7:12533 doi: 10.1038/ncomms12533 (2016).

## Supplementary Material

Supplementary Information Supplementary Figures 1-7, Supplementary Notes 1-3, Supplementary Methods and Supplementary References.

Supplementary Movie 1 A dynamic nonlinear hologram. The different layers address different polarizations, and the rotation of the input polarization switched between the different layers (images) to form a dynamic picture.

Supplementary Movie 2 Polarization controlled nonlinear metalens - focal distance of 1 mm. The movie depicts the focal region of a metalens specifically designed for a given polarization

Supplementary Movie 3 Polarization controlled nonlinear metalens - focal distance of 0.5mm. The movie depicts the focal region of a metalens specifically designed for a given polarization
