# Supplementary material for: Nonlinear metamaterials for holography
Source: Nat Commun. 2016 Aug 22;7:12533. doi: 10.1038/ncomms12533 (PMC4996937; doi:10.1038/ncomms12533)
Supplement: Supplementary Information — Supplementary Figures 1-7, Supplementary Notes 1-3, Supplementary Methods and Supplementary References. [file ncomms12533-s1.pdf]

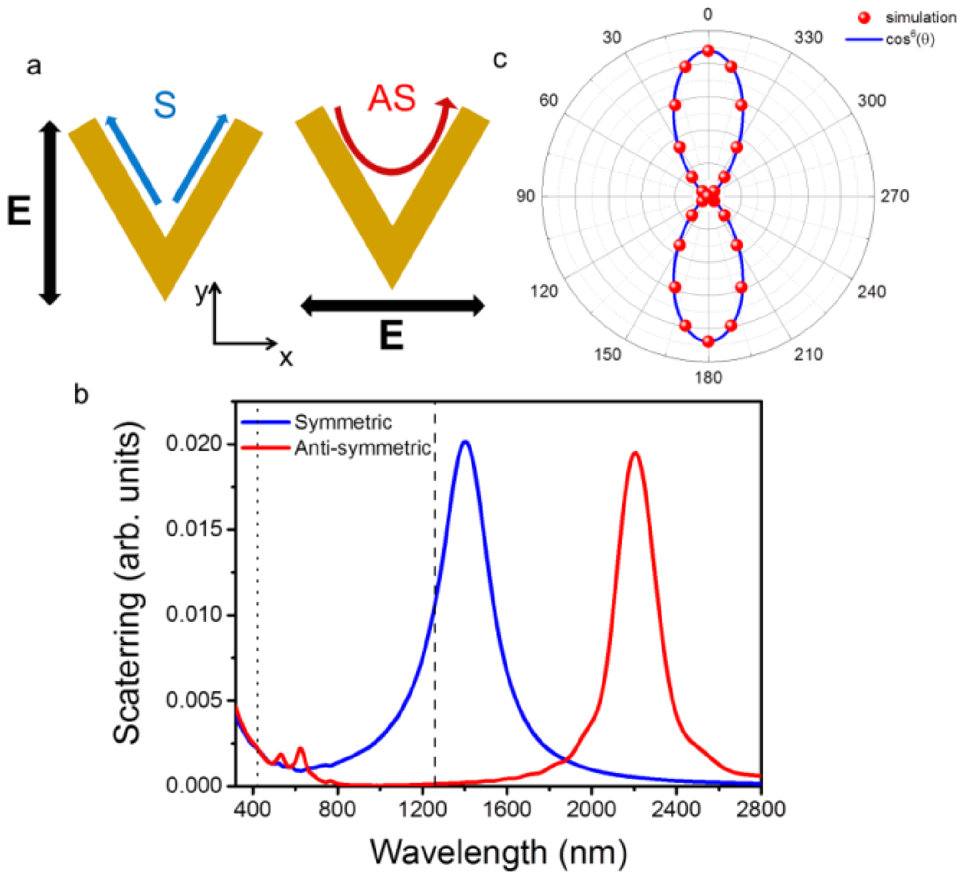

<u>Supplementary Figure 1</u>: Optical Properties of V-shaped Gold Nanoantennas

a) Illustration of the possible plasmonic modes. S- symmetric, AS – antisymmetric. b) Calculated linear scattering spectra of individual antennas with arm length 220 nm and opening angle 60 degrees. C) Calculated polarization dependence of the nonlinear third harmonic signal (red circles) and theoretical curve (blue) for the THG of a dipole.

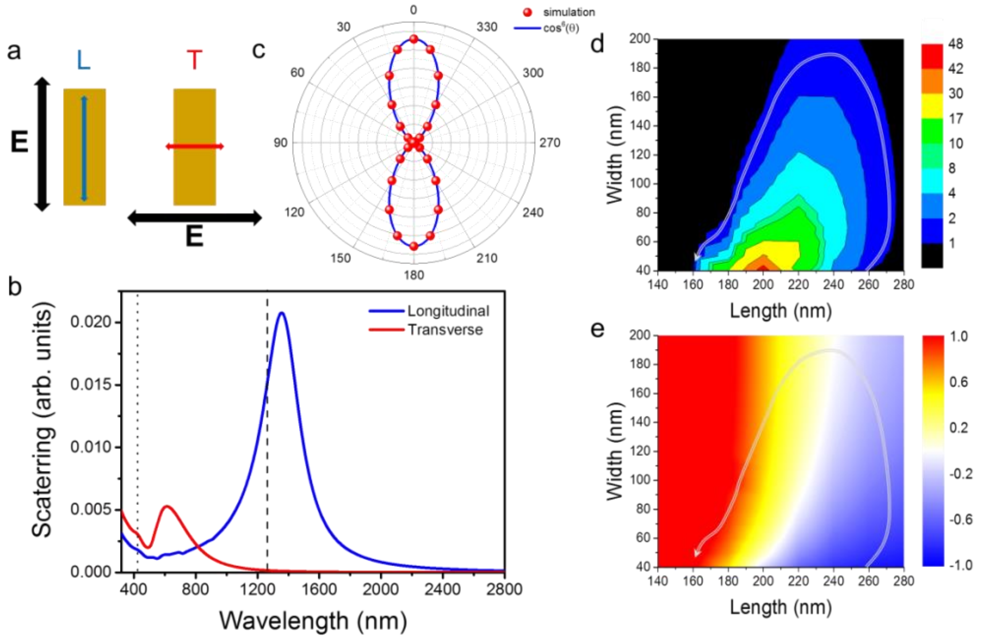

<u>Supplementary Figure 2:</u> Linear and Nonlinear Optical Properties of Gold Nanorods

a) Plasmonic modes. L – Longitudinal, T- transverse. b) Calculated linear scattering spectra. c) Calculated angular dependence of the third harmonic radiation generated by a rod of length L = 200 nm and width W = 80 nm of a dipole (red circles) and a theoretical fit to dipolar radiation (blue curve) d) Amplitude and e) phase map of the third harmonic signal generated by gold nanorods of varying dimensions.

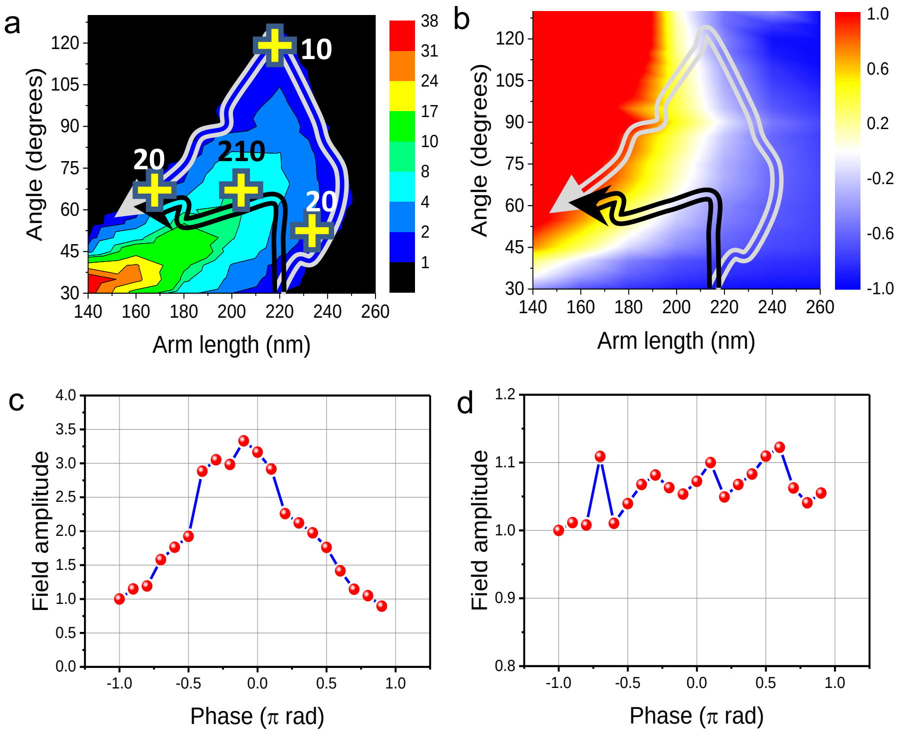

<u>Supplementary Figure 3:</u> Amplitude and Phase for V-shaped antennas

a) Amplitude and b) phase maps for V-shaped antennas, with two different paths and extinction ratio. The amplitude variation as a function of the phase angle are shown for the black path in c) and for the white path in d). The extinction ratios for the perpendicular input polarization for several antennas is marked as the + sign with the relevant local values are marked in a). The larger amplitude and the much larger extinction ratio led us to use the black path.

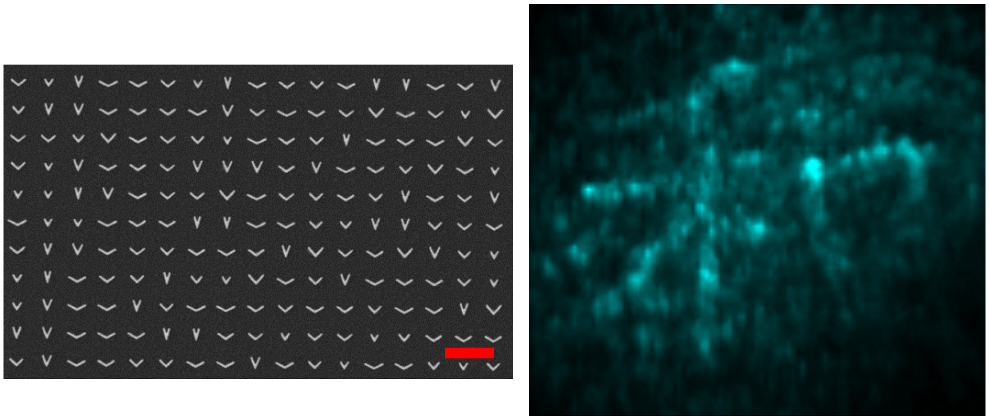

<u>Supplementary Figure 4:</u> Sample hologram along a different path

Sample hologram generated along the white path shown in Supplementary Figure 3a,b with smaller amplitude variations, but significant lower THG enhancement. Note the lower quality when compared to the same image (main text Figure 5c) when generated along the black path, even as part of a multilayer composite structure.

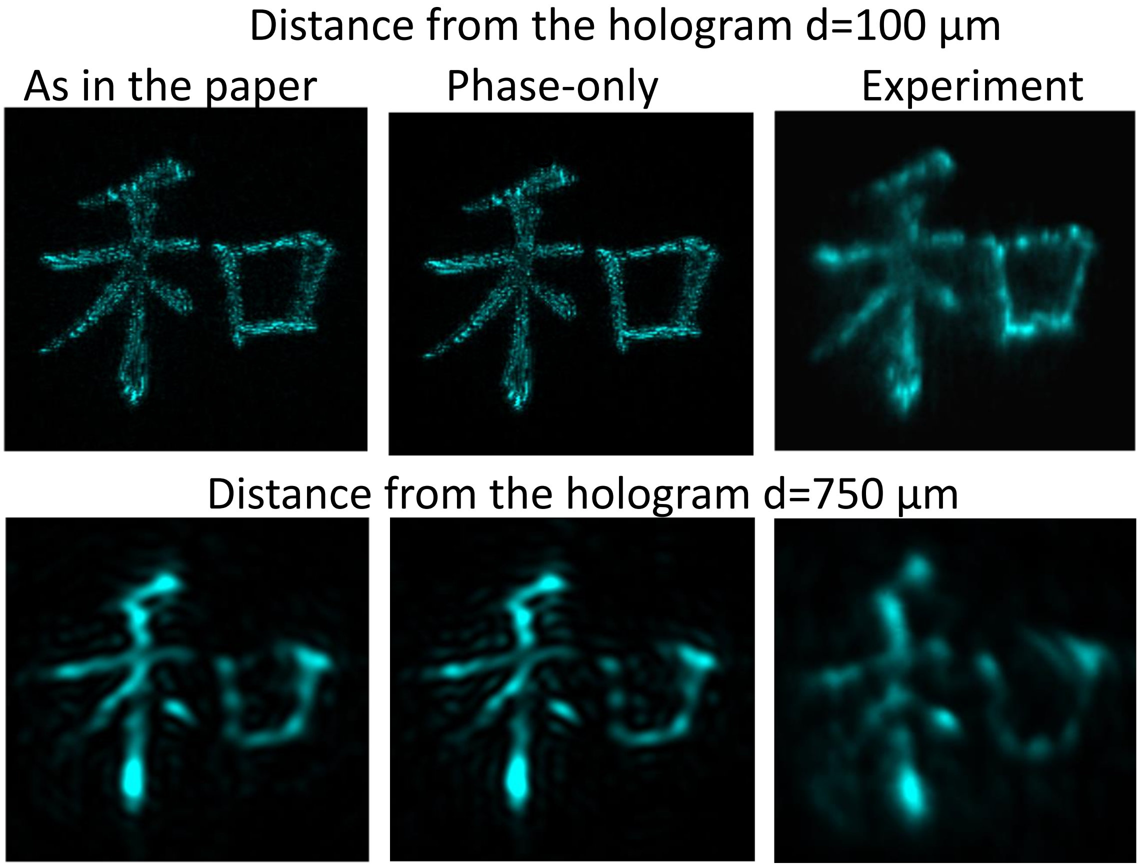

<u>Supplementary Figure 5:</u> Calculated and Measured Holograms

Calculated holograms as in the main text (left), phase only (center) and experimental (right) for two different projection distances: 100µm and 750 µm from the surface. Note the very minimal differences between the pure phase holograms and the calculated one for both phase and amplitude changes as used in the experiment, and the good fidelity of the observed ones.

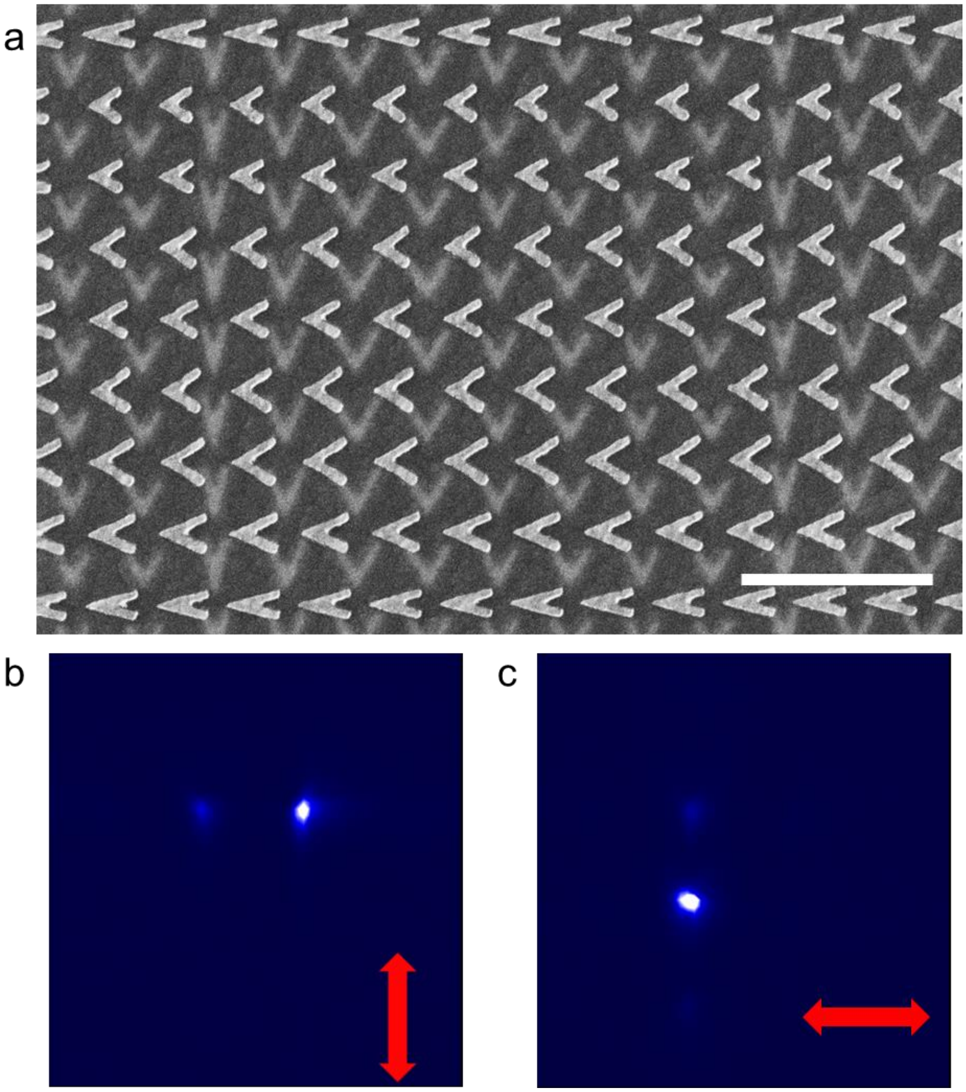

<u>Supplementary Figure 6:</u> Two-layer polarization sensitive nonlinear blazed grating

a) SEM picture of the two layers, The scale bar is 1 micron. b) and c) diffracted beams for the two orthogonal polarizations

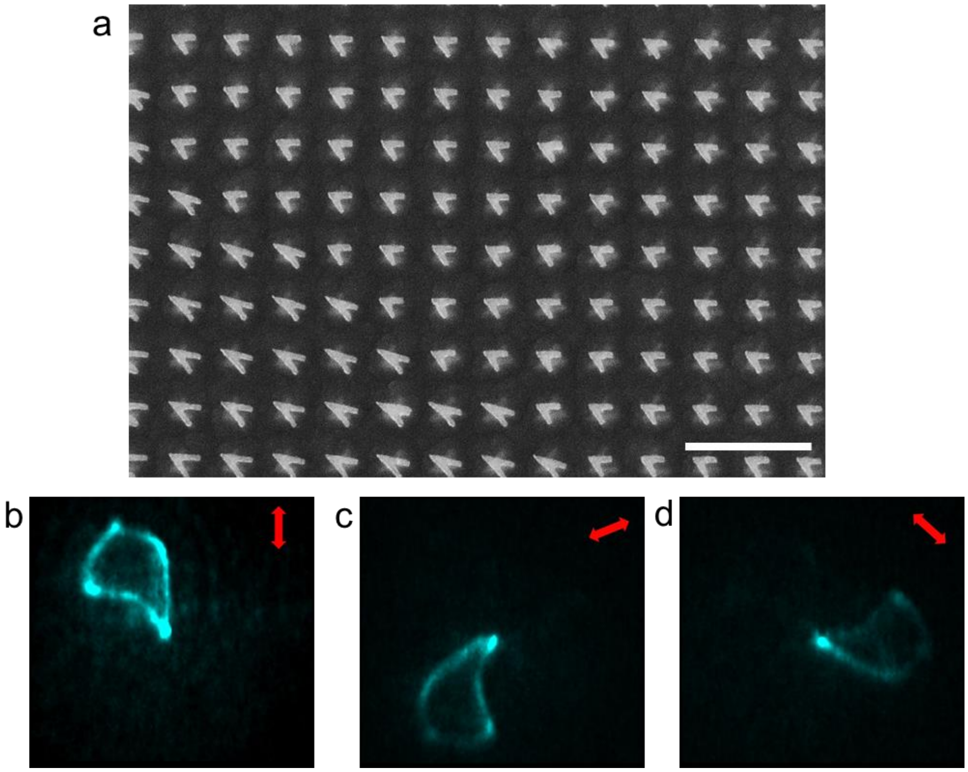

<u>Supplementary Figure 7:</u> Three Layer Dynamic Nonlinear Hologram

a) SEM image of a section of the third layer of the device. The scale bar is 1 μm. b)-d) Holographic NL image for three different polarization states of the fundamental beam. For each polarization state, only one of the layers of the metamaterial is active. A fan-shaped movie can be played when the polarization is continuously rotated.

<u>Supplementary Note 1:</u> Optical Properties of V-shaped and Rod Antennas

The linear optical properties of gold V-shaped nanoantennas were discussed in detail in Supplementary References. [1, 2]. These nanoantennas show two different dipolar plasmonic resonances corresponding to possible modes of oscillation of the electric charges, as illustrated in Supplementary Figure 1. In the symmetric mode, when the light is polarized along the y-axis, the electric charges oscillate symmetrically along each individual arm of the antennas. For light polarized perpendicular to the y-axis, the antisymmetric mode prevails and the electron density switches from one arm to the other. For the nanoantennas used in our holograms, the symmetric mode is blue shifted with respect to the antisymmetric mode. To span the full 2π radians phase shift at the third harmonic frequency, the symmetric resonance is tuned around the frequency of the fundamental beam by controlling the opening angle and the length of the arms. Shorter arms and wider opening angles shift the symmetric resonance to the blue. The antisymmetric mode is also affected by the geometry of the antennas, but it is always detuned with respect to the fundamental beam frequency for the antennas used in our holograms. Therefore, its contribution is negligible to nonlinear signal, as shown in the polar plot in Supplementary Figure 1c.

Similar to V-shaped nanoantennas, gold nanorods are also able capable of imparting a phase shift on the nonlinear signal by more than 2π radians. As shown in Supplementary Figure 2, there are two dipolar modes. The longitudinal mode is redshifted and the charges oscillate along the long axis of the nanorod. The blue-shifted transverse mode is excited by light polarized along the short axis. Amplitude and phase of the nonlinear signal can be controlled by changing the width and the length of the rods. The longitudinal mode is tuned around the laser frequency and depends mainly on the length of the rod. The transverse mode is tuned in the visible and in the near-infrared by changing the width. As shown in Supplementary Figure 2, the transverse mode can be in resonance with the nonlinear signal and may affect the performance of multilayer devices, by imparting a linear phase shift to the harmonic radiation generated in another layer or increasing absorption of the signal. In V-shaped antennas, however the nonlinear signal is always detuned with respect to the antisymmetric mode. Therefore in our holograms we chose to work with the V-shaped antennas.

Supplementary Note 2: Selection of the Optimal Path

There are several possible ways to record the optical information in a hologram [3]. The best fidelity in the reconstruction of an object is attained by a hologram which contains the full information of the complex field in each pixel, i.e. the phase and amplitude. However, phase-only holograms can still reconstruct images with good fidelity that are brighter than for amplitude and phase holograms. Our holograms contain the correct local phase of third harmonic signal as calculated by the point-source method. In order to cover the entire range of 2π radians, a specific path should be followed within the phase map, and the proper geometry should be selected for each antenna. The best path for a phase only hologram would be one without any (or with minimal) amplitude variations. Supplementary Figure 3 depicts two possible paths – one with minimal variation of the amplitude, but with relatively weak response, and one with larger response, but with larger amplitude variations as well. A phase only hologram can be built by using antennas along the white path and with larger opening angles. The images, however, are less bright than those attained for nanoantennas along the higher intensity black path.

To test the quality of holograms generated under the conditions of the white path (namely lower enhancement but with more uniform amplitude), we generated several such holograms, but unfortunately, generally the quality was not as good. An example is shown in Supplementary Figure 4, which is to be compared to the image at Figure 5c in the main text:

In phase holograms, the full information is embedded in the phase of the individual elements, and the amplitude is assumed to be constant over all elements. For our holograms it is not possible to completely separate the two parameters, and as discussed above, the optimal path does include amplitude variation. To demonstrate the fidelity of our holograms with the amplitude variations, we calculated (Supplementary Figure 5a) a hologram for the parameters of our experiment (with amplitude variations as given in Supplementary Figure 3) and compared it to a hologram with identical phases, but with a constant amplitude (Supplementary Figure 5b) , without any detectable deterioration in quality, and for further comparison, the experimental result (Supplementary Figure 5c).

Supplementary Note 3: **Two Layer Polarization Sensitive Blazed Gratings**

We designed a two-layer NL blazed grating which steers the TH beam into different planes. The SEM image is shown in (Supplementary Figure 6a). The scale bar is 1 μm. The top and bottom layers were shifted on purpose. In Supplementary Figure 6b-c, we show a k-space projection of the NL signal diffracted by the blazed gratings onto a CCD camera. An incoming y-polarized fundamental beam steers the third-harmonic along the horizontal direction (Supplementary Figure 6b), while an x-polarized incoming beam generates a TH signal which is steered along the vertical direction (Supplementary Figure 6c). Within a single unit cell, the 2π phase shift necessary to build an efficient blazed grating can be achieved by the nonlinear nanoantennas as shown in Figure 1d in the main text. The zeroth order of diffraction as well as the negative and higher orders are much weaker than the 1st order, which indicates that the phase shift from cell to cell is close to 2π as predicted by NL-FDTD calculations. The beam steering angle measured after calibration is 8.25 ± 0.30 degrees. As discussed in [4], the beam steering angle can be obtained analytically according to the anomalous phase matching condition, which for THG can be expressed as $\boldsymbol{k}_{THG}^{a} = 3\boldsymbol{k}_1 + d\Phi_{THG}/dx$, where $\boldsymbol{k}_1$ is the wavevector of the fundamental beam and $d\Phi_{THG}/dx$ is the phase gradient of the third-harmonic beam along the interface. The anomalous phase matching condition is a manifestation of momentum exchange between the interface and all beams participating in the nonlinear process. For our blazed grating, the angle calculated according to the anomalous phase matching condition is 8.10 degrees, in good agreement with the experimental angle. The beam steering and light focusing capabilities of NL phase control may be a strategy to spatially filter the fundamental and nonlinear beams, and can be useful in nonlinear spectroscopy such as CARS for example.

Supplementary Methods: Sample Fabrication

The samples were fabricated using multilayer e-beam lithography. A 0.15 mm thick borosilicate glass microscope coverslip was used as the substrate. To avoid electron charging on the substrate during SEM, a 3 nm thick chromium layer was initially deposited on the substrate by e-beam evaporation (deposition rate – 0.5 angstroms/s). A 180 nm silica layer was grown by PECVD on top of the Cr film. In the PECVD process, a mixture of gases SiH_4He(5%-95%)/N_2O/N_2 was used at the respective flows 750/1250/400 sccm. The upper and lower electrodes temperatures were kept at 200 °C and 300 °C respectively, and the total gas pressure at 1200 mTorr, resulting in an average deposition rate of 25 angstroms/s. A 125 nm thick e-beam resist (950k PMMA A2) was spin coated (spin rate 1100 rpm) and pre-baked at 180 °C for 90 s. The patterns were exposed by e-beam lithography using accelerating voltage of 30kV and a beam current of 30pA, at a dose of 400 $\mu C/cm^2$. The resist was then developed by MIBK:IPA (1:3) (30 s) and cleaned with isopropyl alcohol (30 s). In order to fabricate a nearly flat patterned surface of gold antennas, the silica pattern is etched via Inductively Coupled Plasma (CF_4 gas, 50 sccm flow, 400W ICP Power and 50 W platen power) for 20 s. A 2 nm thick chromium adhesion layer (evaporation rate – 0.5 angstroms/s) and a 30 nm thick gold layer (evaporation rate 0.7 angstroms/s) were deposited on the patterns by e-beam evaporation. The resist is then lifted off in acetone in an ultrasonic bath. A 180 nm thick silica layer is deposited on the metallic pattern by PECVD and the entire fabrication process is repeated for the additional layers.

While not required for the current holograms, the process described above enables the fabrication of multilayer devices with high stamping accuracy between the layers. Several conditions have to be fulfilled in order to achieve overlay accuracy on the nm scale. The single most important factor leading to low overlay accuracy is the spatial drift of the electron beam during fabrication, potentially arising from several sources. Both system's design and room conditions are optimized to minimize the drift. The optical column is carefully shielded to prevent interferences from stray electro-magnetic fields. The room is also (partially) shielded from electro-magnetic fields and the environmental temperature stability of the room is better than +/- 0.4 degrees Celsius. To exclude residual drifts, the sample was loaded into the system's chamber **10 hours** before exposure, so that thermal equilibrium was reached. Under these conditions, we were able

achieve high beam position stability (<10 nm/5min), high beam current stability (0.5%/hour) and highly accurate marker registration.

For the multilayer fabrication, 4 alignment marks are positioned at each corner of the write-field. Since the alignment marks are defined by deflected beam, a small write field of 100 micron was chosen to avoid error in beam positioning due to deflection of the beam. Automatic and manual mark recognition with small pixel size is performed. During the processing of a layer, the alignment marks might deteriorate and be less visible during the next stages of lithography due to the dielectric film between the layers. Therefore, every layer is exposed with alignment marks as well, which are clearly visible for the next layer. With the above conditions the e-beam stays well aligned with negligible drift within the exposure time of each layer, and good overlay accuracy is maintained. The resulting overlay accuracy in our samples was <15 nm>.

In our multilayer fabrication, each successive layer is buried in a silica isolation layer to form a flat surface. This approach enables flexibility in the use of variety of materials. The ability to control the properties of the dielectric layer such as thickness, roughness, refractive index, transparency and reflectance by choosing suitable material, deposition technique and deposition parameters enables tuning the optical parameters of the system and control over the optical properties of multilayer metamaterial. Thus, the optical properties of PECVD grown films are strongly dependent on the plasma conditions and can be controlled. Moreover, Atomic Layer Deposition (ALD) technique can also be used for very accurate thickness control of very thin films down to very few molecular layers.

By choosing the right material and deposition technique for the spacer, we can tune the resonance of each plasmonic layer. Furthermore, controlling the vertical coupling between adjacent layers by designing very thin dielectric spacers open up possibilities to design and fabricate real three dimensional plasmonic elements. The combination of a wide range of materials, the ability to control the layers and the spacers and the very high stamping accuracy open up opportunities for the design of a variety of 3D plasmonic metasurfaces for many applications.

Supplementary References:

[1] N. F. Yu, P. Genevet, M. A. Kats, F. Aieta, J. P. Tetienne, F. Capasso*, et al.*, "Light Propagation with Phase Discontinuities: Generalized Laws of Reflection and Refraction," *Science,* vol. 334, pp. 333-337, Oct 21 2011.

[2] M. A. Kats, P. Genevet, G. Aoust, N. F. Yu, R. Blanchard, F. Aieta*, et al.*, "Giant birefringence in optical antenna arrays with widely tailorable optical anisotropy," *Proceedings of the National Academy of Sciences of the United States of America,* vol. 109, pp. 12364-12368, Jul 31 2012.

[3] J. Goodman, *Introduction to Fourier Optics*, 3 ed.: Roberts and Company Publishers, 2004.

[4] E. Almeida, O. Bitton, and Y. Prior, "Nonlinear Metamaterials for Holography," *arXiv1512.07899,* 2015.
